# Association between intraoperative computed tomography navigation system and incidence of surgical site infection in patients with spinal surgeries: a retrospective analysis

**DOI:** 10.1186/s13018-022-02936-6

**Published:** 2022-01-29

**Authors:** Gentaro Kumagai, Kanichiro Wada, Sunao Tanaka, Toru Asari, Yohshiro Nitobe, Yasuyuki Ishibashi

**Affiliations:** Department of Orthopaedic Surgery, Hirosaki University Graduate School of Medicine, 5 Zaifu-cho, Hirosaki, Aomori 036-8562 Japan

**Keywords:** Spinal surgery, Intraoperative fluoroscopy, Intraoperative CT, Surgical site infection

## Abstract

**Purpose:**

Although the use of intraoperative computed tomography (CT)-based navigation systems is unlikely to cause intraoperative contamination more than the use of intraoperative fluoroscopy, the association between intraoperative CT/navigation and surgical site infections (SSIs) remains unclear. We investigated the incidence of SSIs and the association between intraoperative CT/navigation and SSIs for spinal surgeries.

**Methods:**

Of the 512 patients who underwent spinal surgery between April 2016 and December 2020, 304 underwent C-arm intraoperative fluoroscopy and/or Medtronic O-arm intraoperative CT/navigation system. We investigated the incidence of SSIs in patients with four techniques; no intraoperative imaging C-arm only, O-arm only, and both O- and C-arm used. Multivariate logistic analyses were conducted using the prevalence of SSIs as the dependent variable. The independent variables were age, sex, and potential confounders including preoperative Japanese Orthopaedic Association (JOA) score, use of instrumentation, C-arm, and/or O-arm.

**Results:**

The incidence of the SSIs in patients with no imaging, C-arm only, O-arm only, and both modalities used was 1.9%, 7.3%, 4.7%, and 8.3%, respectively. There was no significant difference in the incidence of SSIs between the four techniques. Multivariate logistic analyses showed a significant correlation between the prevalence of SSI and JOA scores (odds ratio, 0.878; 95% CI 0.759–0.990) and use of instrumentation (odds ratio, 6.241; 95% CI 1.113–34.985), but not use of O-arm.

**Conclusions:**

The incidence of the SSIs in patients with only O-arm used was 4.7%. Preoperative clinical status and use of instrumentation, but not use of the O-arm, were associated with SSIs after spinal surgeries.

## Background

Surgical site infections (SSIs) result in increased patient morbidity, mortality, and healthcare costs. The incidence of SSIs in spinal surgery varies widely; a large database showed an incidence of 0.72% for laminectomy with no risk factors to 8.7% for refusion of the spine in patients with three risk factors [1]. The etiology of postoperative SSIs is multifactorial and is often related to a combination of preoperative, intraoperative, and postoperative factors. Instrumentation surgery was reported to be a significant risk factor for SSIs [2]. Intraoperative fluoroscopy and computed tomography (CT)-based navigation are routinely used in the operating room for a variety of spinal instrumentation surgeries. Previous studies have shown that the accuracy of pedicle screw placement using C-arm fluoroscopy-based image-guided techniques was higher than that of manual techniques [3, 4]. Recent studies have shown that intraoperative CT-based guidance improves the accuracy of pedicle screw placement more than does standard fluoroscopy [5, 6]. 3D preoperative planning and navigation also improve bone tumor resection [7] and the gutter position of cervical laminoplasty [8]. Moreover, intraoperative CT-based imaging reduces radiation dose to patients [9]. Whichever imaging systems guide the spinal instrumentation surgeries, the intraoperative fluoroscopy and CT devices need to be kept sterile while imaging with fluoroscopy and CT scanning. Although the intraoperative CT/navigation system is unlikely to cause intraoperative contamination more than does intraoperative fluoroscopy, the association between intraoperative CT/navigation and SSIs remains unclear. The study investigated the incidence and associated factors of SSIs after using Medtronic O-arm CT/navigation system including other potential confounders for spinal surgeries.

## Methods

### Patients

**Fig. 1:**
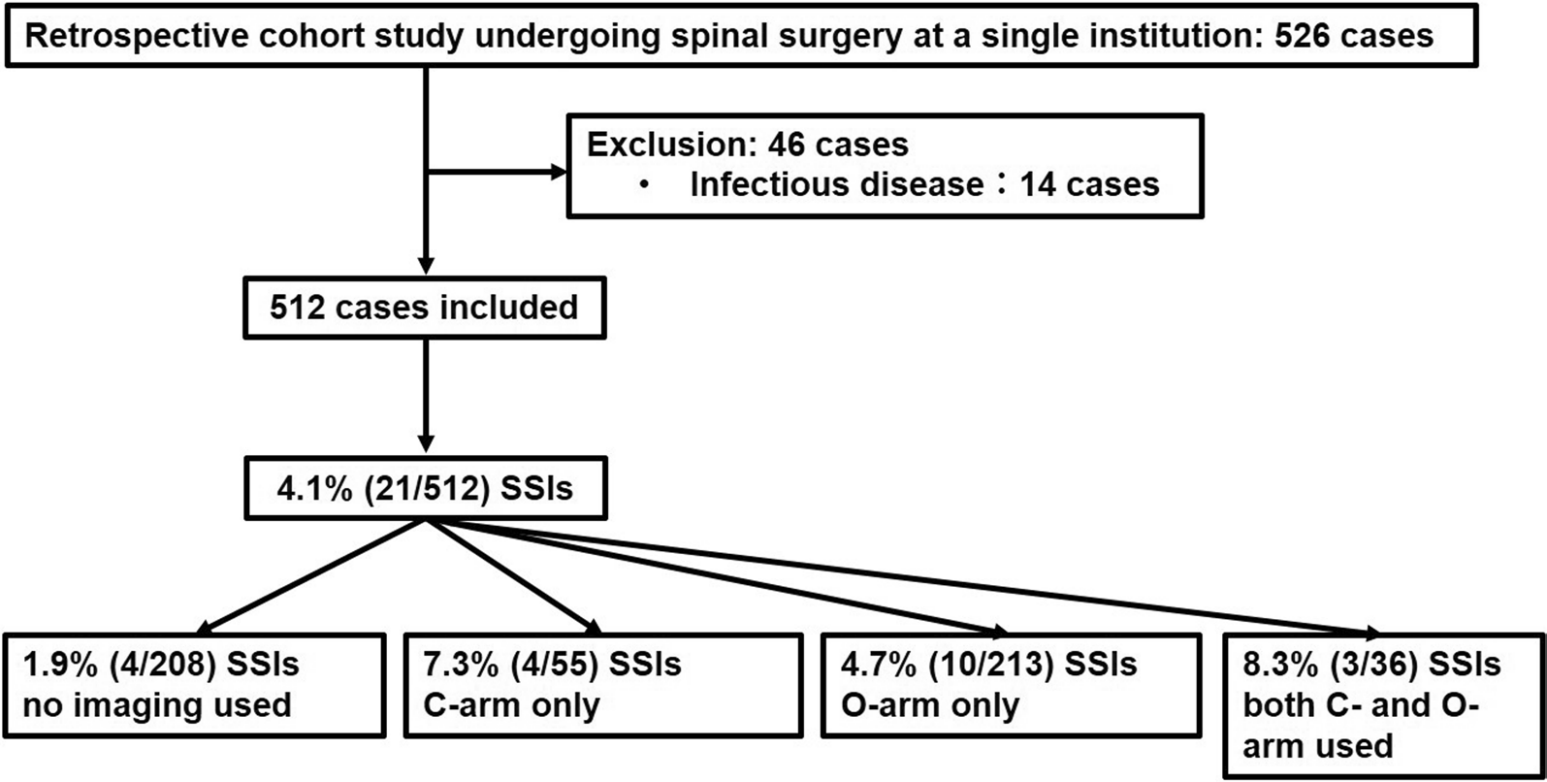
Flow diagram in this study. The subjects were 512 patients who underwent posterior spine surgery at our department. The incidence of the SSIs in all patients was 4.1%. The incidence of the SSIs when no imaging, C-arm only, O-arm only, or both modalities were used was 1.9%, 7.3%, 4.7%, and 8.3%, respectively

Overall, 526 consecutive patients who underwent spine surgery between April 2016 and December 2020 were enrolled in the study. Patients with preoperative pyogenic spondylitis and septic wound conditions were excluded. Finally, 512 patients (242 men and 270 women) were included (Fig. 1) and a retrospective analysis of their surgeries and outcomes was completed. Diagnoses are presented in Table 1. The mean age was 64.0 years old. Fifty-five patients were treated using C-arm intraoperative fluoroscopy. For 213 patients, intraoperative CT (Medtronic Sofamor Daneck, Memphis, TN, US) and a navigation StealthStation system (Medtronic Sofamor Danek) were used. Thirty-six patients were treated using both the C-arm and O-arm intraoperatively. Two-hundred eight patients were treated without intraoperative imaging. Intraoperative CT and a navigation StealthStation system were used in cases of insertion of a pedicle screw (PS) [10], cervical laminoplasty [8], and resection of bone tumor and decompression. In cases of posterior lumbar interbody fusion, a navigation StealthStation system was used for insertion of the pedicle screw after intraoperative CT examination, and C-arm for the lumbar interbody fusion technique. All patients provided written informed consent before assessment. This research report has been approved by the IRB of the authors’ affiliated institution.

**Table 1:** Characteristics, diagnosis, and surgical information in each technique

	Intraoperative imaging information			
	No imaging (*n* = 208)	C-arm only (*n* = 55)	O-arm only (*n* = 213)	Both arms (*n* = 36)
Characteristics				
Age^a^, years	65.00 (54.25–75.00)	57.00 (16.00–66.25)	63.00 (21.0–72.00)	64.50 (58.00–71.50)
Female sex, N (%)	103 (49.5)	27 (49.1)	117 (54.9)	23 (63.9)
BMI^a^, kg/m^2^	24.52 (21.87–27.84)	22.00 (18.76–26.54)	22.97 (20.37–26.11)	25.32 (22.17–28.60)
Obesity, N (%)	102 (49.0)	17 (30.9)	71 (33.3)	20 (55.6)
Hypertension, N (%)	79 (38.0)	13 (23.6)	70 (32.9)	15 (41.7)
Diabetes, N (%)	34 (16.3)	9 (16.4)	49 (20.0)	10 (27.8)
Hyperlipidemia, N (%)	11 (5.3)	3 (5.5)	11 (5.2)	3 (8.3)
Chronic renal failure, N (%)	12 (5.8)	0 (0)	3 (1.4)	2 (5.6)
Steroid use, N (%)	3 (1.4)	1 (1.8)	8 (3.8)	6 (16.7)
Smoking, N (%)	10 (4.8)	5 (9.0)	12 (5.6)	0 (0)
ASA-PS ≥ 3, N (%)	53 (25.5)	18 (32.7)	70 (32.9)	9 (25.0)
JOA score^a^	13.50 (11.50–15.00)	15.00 (11.00–17.00)	14.00 (10.50–17.00)	13.25 (11.75–15.00)
Diagnosis				
Trauma				
Cervical, N (%)	1 (0.5)	2 (3.6)	3 (1.4)	0 (0)
Thoracic, lumbar, N (%)	3 (1.4)	3 (5.5)	7 (3.3)	0 (0)
Degenerative disease				
Cervical, N (%)	38 (18.3)	9 (16.4)	107 (50.2)	2 (5.6)
Thoracic and lumbar, N (%)	95 (45.7)	19 (34.5)	13 (6.1)	31 (86.1)
Spinal cord tumor, N (%)	52 (25)	1 (1.8)	7 (3.3)	0 (0)
Spinal deformity, N (%)	6 (2.9)	17 (30.9)	64 (30.0)	3
RA, N (%)	1 (0.5)	1 (1.8)	7 (3.3)	0 (0)
DSA, N (%)	0 (0)	0 (0)	3 (1.4)	0 (0)
Metastatic disease, N (%)	1 (0.5)	0 (0)	2 (0.9)	0 (0)
Others, N (%)	11 (5.3)	3 (5.5)	0 (0)	0 (0)
Surgical information				
Instrumentation, N (%)	5 (2.4)	51 (92.7)	134 (62.9)	36 (100)
Posterior surgery, N (%)	208 (100)	54 (98.2)	210 (98.6)	34 (94.4)
Number of surgical levels^a^	2.00 (1.00–3.00)	4.00 (1.00– 7.00)	5.00 (4.00 – 8.00)	2.00 (1.00 – 3.00)
Duration of surgery^a^	176.50 (123.00–257.00)	266.00 (198.00 – 352.00)	262.00 (181.50– 366.00)	269.50 (225.50– 373.20)
Blood loss (mL)^a^	95.00 (30.00–217.50	350.00 (150.00–810.00)	250.00 (80.00–580.00)	240.00 (122.25–560.00)

BMI, body mass index; ASA-PS, American Society of Anesthesiologists Physical Status; JOA, Japanese Orthopaedic Association., RA: rheumatoid arthritis, DSA: destructive spondyloarthropathy

^a^Values are presented as median value and interquartile (IQR)

### Protocol for the prevention of SSIs

Peri-operative management for the prevention of SSIs was performed as previously described [11]. Patients who were smokers were instructed to cease smoking for > 4 weeks before surgery. Patients with diabetes and hemoglobin A1c levels of > 7% underwent glycemic control before surgery. The blood glucose levels of patients over the age of 60 years were controlled, on a sliding scale, for 1–2 weeks after surgery until blood glucose levels were normal. Prophylactic antibiotics were administered via intravenous drip infusion only during the preoperative and intraoperative periods in all consecutive spine surgeries. Cefazolin (based on body weight) was administered as the first choice unless the patient had a history of significant allergies, such as anaphylactic shock, systemic skin eruption, or toxic liver dysfunction. If necessary, the skin hair in the surgical area was removed with clippers in the operating room before surgery. Preoperative antibiotics were administered within 30 min before skin incision. The surface of the surgical site was cleaned using chlorhexidine in the preoperative period and was immediately coated with iodine drape. Glucodine was used as an additional skin preparation solution from June 2018. Intraoperatively, an additional dose of antibiotics was administered every four hours. If the operation was completed within 4 h, no additional antimicrobial agent was administered. The surgical site was irrigated using saline solution only as often as possible during the surgery, and a large amount of saline solution was used before closing the surgical site. In the postoperative period, the surgical site was managed with continuous negative pressure suction drainage until 48 h after surgery.

The identification of an SSI involves the interpretation of clinical and laboratory findings. Clinical signs included purulent exudate, surrounding erythema, and wound fluctuance. Laboratory data included prolonged elevation in the values of white blood cells, C-reactive protein, and erythrocyte sedimentation rate. Superficial SSIs involve only the skin or subcutaneous tissue of the incision. Deep SSIs involve the fascia and muscle layers of the incision. An SSI was defined as infection occurring within 30 days after the operation if no implant was left in place, or within one year if the implant was in place and the infection appeared to be related to the surgery. Cefazolin was administered immediately as the first choice if the patient had an SSI. In patients with SSIs, if the bacteria were not sensitive to cefazolin, sensitive antibiotics were administered. All patients could be assessed for any additional SSIs that occurred within one year.

### Sterile drape

**Fig. 2:**
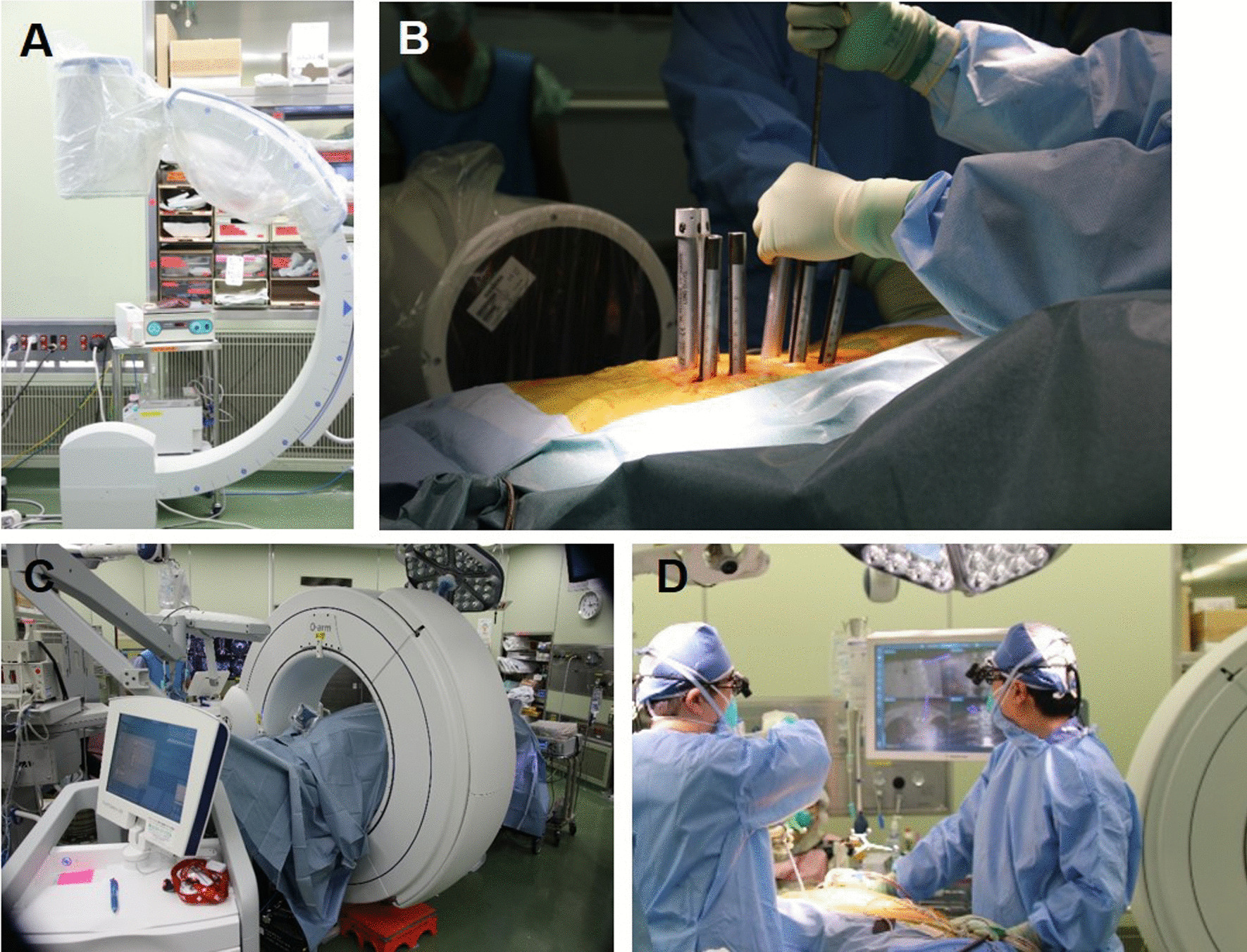
A sterile procedure of A-arm and O-arm/navigation. C-arm fitted with a sterile drape (**A**), positioned to acquire AP view and was covered with sterile drapes lateral view (**B**). The **s**urgical area was covered with sterile drapes while scanning using the O-arm (**C**). Surgeons used navigation system (**D**). AP, anteroposterior

All patients were positioned prone on a radiolucent Jackson spinal table (Mizuho OSI, Union City, CA, USA). For patients with whom the C-arm was used, it was fitted with a sterile drape by scrub doctors (Fig. 2A), and the standard aseptic technique was maintained throughout the surgery by the radiology technician operating the C-arm (Fig. 2B). For patients with whom the O-arm was used, the surgical area was covered with sterile drapes while scanning with the O-arm (Fig. 2C). After scanning, surgeons performed the surgical procedure using a navigation system (Fig. 2D).

### Evaluation of clinical outcomes

The severity of clinical symptoms was evaluated using the Japanese Orthopaedic Association (JOA) score to assess cervical myelopathy prior to surgery in each group. This scale consists of six domain scores (motor dysfunction in the upper extremities, motor dysfunction in the lower extremities, sensory function in the upper extremities, sensory function in the trunk, sensory function in the lower extremities, and bladder function) scaled from 0 to 4, 4, 2, 2, 2, and 3, respectively, with a minimum total score of 0 and a maximum total score of 17.

### Statistical analyses

SPSS version 24 (SPSS Inc., Chicago, IL, USA) was used for data input and statistical calculations. The median and interquartile range (IQR) were calculated for continuous variables. The percentage and 95% confidence interval (CI) were calculated for categorical variables. The Mann–Whitney U test and Chi-square test were used to compare the characteristics between males and females. We also compared the conditions with and without SSI for potential risk factors using the Mann–Whitney U test and Chi-square test, respectively. The potential risk factors included age, sex, body mass index (BMI), obesity (BMI > 25), presence of hypertension and diabetes, steroid use, smoking, American Society of Anesthesiologists physical status (ASA-PS) of ≥ 3, JOA score, duration of surgery, and blood loss as known risk factors of SSIs [12]. Independent risk factors for SSIs were identified from multivariate logistic regression analyses, conducted using the prevalence of SSI as the dependent variable in each group. Independent variables with age, sex, BMI, C-arm and/or O-arm, and potential confounders which had a P-value of < 0.05 in the univariate analysis, were eligible for inclusion in the multivariate models. A P-value of < 0.05 was considered statistically significant.

## Results

### Characteristics, diagnosis, and surgical information in each technique

Table 1 shows the patients’ characteristics, diagnosis, and surgical information in each technique. The frequency of steroid use was the highest in both C-arm and O-arm (16.7%). The most common diagnosis in patients who required no imaging, C-arm only, or both C-arm and O-arm imaging was degenerative disease in the thoracic and lumbar spine (45.7%, 34.5%, and 86.1%, respectively). The most common diagnosis in patients with only O-arm imaging was degenerative disease in the cervical spine (50.2%). The prevalence of instrumentation surgery was low in patients not requiring imaging (2.4%) and was high in patients imaged with the C-arm and/or O-arm (92.7% and 62.9%). The number of surgical levels in patients imaged using only the O-arm was the highest among all techniques. The duration of surgery was lowest in patients for whom intraoperative imaging was not used. Blood loss was highest in patients imaged with the C-arm only.

### Characteristics of patients in each gender

Table 2 shows the patients’ characteristics related to age, BMI, obesity, medical history, smoking, steroid use, ASA-PS ≥ 3, and JOA score for each sex. The BMI in males was significantly higher than that in females (*P* = 0.027). The prevalence of hypertension and diabetes, and habit of smoking in males was significantly higher than that in females respectively (*P* = 0.009, 0.003, and 0.005). No significant differences were found for age, the prevalence of hyperlipidemia and chronic renal failure, steroid use, ASA-PS ≥ 3, and JOA score.

**Table 2:** Characteristics of patients in each gender

	All (*n* = 512)	Male (*n* = 242)	Female (*n* = 210)	*P* value^b^
Age^a^, years	64.00 (46.5–73.0)	64.00 (51.0–72.0)	63.0 (31.5–72.0)	0.707
BMI^a^, kg/m^2^	23.87 (20.73–27.15)	24.40 (21.58–27.00)	22.98 (19.83–27.15)	0.027*
Obesity, N (%)	210 (46.5)	114 (47.1)	96 (35.6)	0.009^#^
Hypertension, N (%)	177 (39.2)	100 (41.3)	77 (28.5)	0.003^#^
Diabetes, N (%)	102 (22.6)	62 (25.6)	40 (14.8)	0.003^#^
Hyperlipidemia, N (%)	28 (6.2)	17 (7.0)	11 (4.1)	0.174
Chronic renal failure, N (%)	25 (5.5)	16 (6.6)	9 (3.3)	0.101
Steroid use, N (%)	18 (4.0)	6 (2.4)	12 (4.4)	0.337
Smoking, N (%)	27 (6.0)	20 (8.3)	7 (2.6)	0.005^#^
ASA-PS ≥ 3, N (%)	150 (33.2)	80 (33.1)	70 (25.9)	0.081
JOA score^a^	13.5 (11.0–16.0)	13.5 (10.0–15.0)	14.0 (11.5–17.0)	0.264

BMI, body mass index; ASA-PS, American Society of Anesthesiologists Physical Status; JOA, Japanese Orthopaedic Association.

^a^Values are presented as median value and interquartile (IQR)

^b^Significant differences (*P* < .05) between values for men and women were calculated by *Mann–Whitney U or ^#^Chi-square test

### Incidence of SSIs and associated factors

The incidence of SSIs in all patients was 4.1% (21 cases) (Fig. 1). The incidence of the SSIs without imaging, or with C-arm only, O-arm only, and both C- and O-arm was 1.9%, 7.3%, 4.7%, and 8.3%, respectively (Fig. 1). There was no significant difference in the incidence of SSIs between the four techniques (*P* = 0.125).

Table 3 shows cases of SSIs in each technique. There was one case of superficial SSI and two cases of deep SSI in patients for whom imaging was not used. There were four cases of superficial SSI in patients imaged only using the C-arm. There were three cases of superficial SSI and seven cases of deep SSI in patients imaged only with the O-arm. There were three cases of superficial SSI in patients imaged with both modalities.. Patients with superficial SSIs were treated only with antibiotics. Patients with deep SSIs were treated using surgical intervention and antibiotics without implant removal. Causative bacteria of SSIs were methicillin-sensitive *coagulase-negative Staphylococci* (MSCNS) and methicillin-resistant *coagulase-negative Staphylococci* (MRCNS) in four cases, methicillin-sensitive *Staphylococcus aureus* (MSSA) in two cases, *Propionibacterium acnes, Enterobacter cloacae*, and *Enterococcus faecalis* in one case each. No bacteria were cultured in five cases, although clinical signs appeared.

**Table 3:** Cases of SSIs in each techniques

Age (years), gender	Comorbidities	Diagnosis	Surgical methods/Instrumentation	Type of SSI*	SSI bacteria	Additional surgery
No intraoperative imaging						
84, female	None	LDH	Herniotomy/No	S	N. D.	No
51, male	Psoriasis	OPLL	Cervical laminoplasty/No	D	*Enterobacter cloacae*	Yes
76, male	Hypertension, malignant lymphoma	LSS	Laminectomy/No	D	*Enterococcus faecalis*	Yes
C-arm only						
56, male	Athetoid cerebral palsy	CSM	Posterior fusion/Yes	S	MSSA	No
66, female	Collagen disease (steroid)	Trauma	Posterior fusion/Yes	S	MSCNS MSSA	No
80, male	Cancer	LSS	Posterior fusion/Yes	S	N. D.	No
78, male	Hypertension, diabetes, stroke	CSM	Posterior fusion/Yes	S	N. D.	No
O-arm only						
65, male	Hypertension, diabetes, stroke	CSM	Posterior fusion/Yes	S	N. D.	No
13, male	Mental retardation	Scoliosis	Posterior fusion/Yes	S	N. D.	No
62, male	Cirrhosis	CSM	Posterior decompression/No	S	*Propionibacterium acnes*	No
72, female	RA	Atlantoaxial subluxation	Posterior fusion/Yes	D	MSCNS	Yes
48, female	Athetoid cerebral palsy	CSM	Posterior fusion/Yes	D	MSCNS	Yes
66, male	OPLL	T12 burst fracture	Posterior fusion/Yes	D	MRCNS	Yes
75, female	Cancer	Pseudotumor of C1	Posterior fusion/Yes	D	MRCNS	Yes
43, female	Obesity	OPLL	Posterior fusion/Yes	D	MSCNS	Yes
40, male	–	Tumor of thoracic spine	Posterior fusion/Yes	D	*Propionibacterium acnes*	Yes
75, female	RA	Atlantoaxial subluxation	Posterior fusion/Yes	D	MRSA	Yes
Both O- and C-arm used						
69, female	Chronic renal failure	LSS	Posterior fusion/Yes	S	MRCNS	No
88, female	Hypertension	Trauma	Posterior fusion/Yes	S	N. D.	No
52, female	Hypertension, diabetes, stroke, asthma	LSS	Posterior fusion/Yes	S	MRCNS	No

SSI, surgical site infection; CSM, cervical spondylotic myelopathy; LSS, Lumbar disc herniation; LDH, lumbar spinal stenosis; OPLL, ossification of posterior longitudinal ligament; MSCNS, methicillin-sensitive coagulase-negative staphylococci; MSSA, methicillin-sensitive *Staphylococcus aureus*; N. D., not detected; *Type of SSI: S, superficial; D, deep SSI

Table 4 shows univariate and multivariate predictors of SSIs in patients after spinal surgeries. The prevalence of steroid use and ASA-PS ≥ 3 in patients with SSI was significantly higher than that in patients with no SSI (*P* = 0.003 and 0.026), respectively (Table 4). The preoperative JOA score was significantly lower in patients with SSI than in patients without SSI (*P* = 0.041) (Table 4). Multivariate logistic analyses showed a significant correlation between the prevalence of SSI and JOA scores (odds ratio, 0.878; 95% confidence interval, 0.759–0.990) and use of instrumentation (odds ratio, 6.241; 95% confidence interval, 1.113–34.985), but not when the O-arm was used (Table 4).

**Table 4:** Univariate and multivariate predictors of SSI in patients after spinal surgeries

Variables	Patients with SSI (*n* = 21)	Patients without SSI (*n* = 491)	*P* value^b^	Multivariate analysis*		
				OR	95% CI	*P* value
Age, years	66.00 (54.00–75.50)	64.00 (46.00–73.00)	0.482	1.018	0.987–1.051	0.260
Female sex	10	260	0.661	0.987	0.381–2.559	0.979
BMI^a^, kg/m^2^	25.00 (21.72–30.23)	23.70 (27.70–27.10)	0.373	1.042	0.947–1.145	0.400
Obesity,	10	200	0.651	–	–	–
Hypertension, N (%)	7	170	1.000	–	–	–
Diabetes, N (%)	3	99	0.780	–	–	–
Hyperlipidemia	1	27	1.000	–	–	–
Chronic renal failure	1	24	1.000	–	–	–
Steroid use	3	15	0.033^#^	3.104	0.677–14.227	0.145
Smoking	0	27	0.618	–	–	–
ASA-PS ≥ 3	11	139	0.026^#^	1.878	0.702–5.025	0.209
JOA score^a^	11.50 (7.00–13.50)	13.20 (11.00–16.00)	0.041*	0.878	0.779–0.990	0.034
C-arm use	4	52	0.271	0.921	0.107–7.921	0.940
O-arm use	10	204	0.654	0.631	0.102–3.886	0.619
Both use	3	33	0.177	0.628	0.064–6.136	0.619
Instrumentation	16	211	0.003^#^	6.241	1.113–34.985	0.037
Number of surgical levels^a^	4.00 (2.00–5.00)	4.00 (2.00–5.00)	0.274	–	–	–
Duration of Surgery, min	266.00 (186.50–367.50)	217.00 (154.25–321.75)	0.123	–	–	–
Blood loss, ml	330.00 (90.00–725.00)	150.00 (50.00–400.00)	0.260	–	–	–

SSI, surgical site infection; OR, odds ratio; BMI, body mass index; ASA-PS, American Society of Anesthesiologists Physical Status; JOA, Japanese Orthopaedic Association. ^a^ Values are presented as median value and interquartile (IQR). ^b^Significant differences (*P* < .05) between values for patients with SSI and without SSI were calculated by *Mann–Whitney U or #Chi-square test

## Discussion

To the best of our knowledge, this is the first study to clarify the incidence of SSIs after using the intraoperative O-arm/navigation system for spinal surgeries. Preoperative clinical status and use of instrumentation, but not intraoperative CT/navigation, was associated with SSIs after spinal surgeries.

The incidence of SSIs in spinal surgery varies widely, and a large database indicated an incidence of 0.72% for laminectomy with no risk factors to 8.7% for refusion of the spine in patients with three risk factors [1]. In the Medicare population, Kurtz et al. reported the rate of SSIs in instrumented patients as 8.5% in primary fusions and 12.2% in refusions [13]. In our SSI prevention protocol, the incidence of SSIs when either the C-arm, O-arm, or both were used together was 7.3%, 4.7%, and 8.3%, respectively, and are similar to those reported in previous studies of instrumented spinal surgeries [1].

Several studies have identified risk factors for SSIs in spine surgery. A recent meta-analysis reported 13 risk factors that were statistically significant [14]. Those that were modifiable through patient selection and optimization included ASA-PS of > 2 (OR 2.27; 95% CI 1.5–3.42), diabetes (OR 2.04; 95% CI 1.69–2.46), obesity (OR 2.21; 95% CI 1.55–2.93), BMI (OR 0.25; 95% CI 0.1–0.4), revision surgery (OR 1.85; 95% CI 1.46–2.34), smoking (OR 1.17; 95% CI 1.03–1.32), urinary tract infection (OR 3.19; 95% CI 1.68–6.06), hypertension (OR 1.67; 95% CI 1.26–2.22), CSF leak (OR 3.22; 95% CI 1.07–9.67), and dural tear (OR 3.01; 95% CI 1.6–5.66). Although age was not a significant factor, other studies have reported that older age was a significant risk factor independent of comorbidities. Additionally, patients with diabetes have been shown to have worse patient-reported outcomes for up to two years after spine surgery [15]. In our study, patients with SSIs did not have diabetes, and diabetes was not a risk factor for SSI. These results raise the real possibility that stringent glycemic control may mitigate the risk of SSIs. Our study suggested the severity of neurological findings as JOA score were associated with SSIs. Therefore, we need to pay attention to prevent SSIs in patients with severe cervical spondylotic myelopathy and spinal cord injury. Moreover, a systematic review showed that 2 of the 6 studies found a statistically significant association between instrumentation and postoperative SSI [12]. Therefore, the author concluded they could not rule out a possible association. Further analysis of the role instrumentation as independent risk factors for SSIs was beyond the scope of this initial study and will be explored in the future.

A common source of contamination in spine surgery is the use of a “C-arm” for intraoperative fluoroscopy. To maintain sterility, a sterile drape is placed over the portion of the machine that will be in close contact with the operating field. Biswas performed a prospective study to assess the sterility of C-arms at the end of 25 spine surgery cases [16]. Five areas of the C-arm were cultured immediately after drape application and at the end of surgery. One location (4%) was culture positive after immediate draping. All drapes were cultured at the end of the procedure, and all areas were contaminated, with the upper areas of the C-arm being most frequently contaminated. The authors recommend that these regions need to be considered nonsterile. The use of intraoperative CT and navigation systems is unlikely to cause intraoperative contamination more than the use of intraoperative fluoroscopy. Since there was no significant difference in the incidence of SSIs between the use of C-arm and O-arm, SSIs may be more associated with factors other than with the surgical environment.

Our study has several limitations. First, in our protocol, surgical methods, type of instrumentation, surgeons, and the number of operating room staff were different between all the techniques. Second, the cultures of the C-arm and O-arm equipment were not examined before and after surgery. Therefore, the mechanisms underlying development of the SSIs remain unclear. Third, the reason for many deep SSI cases (70%) when only is used is unknown. Fourth, we did not evaluate high-risk patients for MRSA colonization and SSI risks. Finally, our sample size for the C-arm group and the group where both images were used was too small.

## Conclusions

The incidence of the SSIs in patients imaged only with the O-arm was 4.7%. Preoperative clinical status and use of instrumentation, but not intraoperative fluoroscopy and CT, were associated with SSIs after spinal surgeries. This findings could provide clinical information of patients undergoing spinal instrumentation surgery.

## Data Availability

The data are available on reasonable request from the corresponding author.

## Abbreviations

SSIs: Surgical site infections
CT: Computed tomography
JOA score: The Japanese Orthopaedic Association score
IQR: Interquartile range
CI: Confidence interval
BMI: Body mass index
ASA-PS: American Society of Anesthesiologists physical status
MSCNS: Methicillin-sensitive *coagulase-negative Staphylococci merican*
MRCNS: Methicillin-resistant *coagulase-negative Staphylococci*
MSSA: Methicillin-sensitive *Staphylococcus aureus*
